# A Proposed Model of Core Competencies for Research Ethics Consultants

**DOI:** 10.1007/s41649-021-00178-y

**Published:** 2021-05-29

**Authors:** Kenji Matsui, Yusuke Inoue, Hiroaki Yanagawa, Tadao Takano

**Affiliations:** 1 Division of Bioethics and Healthcare Law, National Cancer Center Japan, Tokyo, Japan; 2 Department of Public Policy, the Institute of Medical Science, the University of Tokyo, Tokyo, Japan; 3 University Hospital of Tokushima Clinical Trial Center for Developmental Therapeutics, Tokushima, Japan; 4 Clinical Research, Innovation and Education Center, Tohoku University Hospital, Sendai, Japan

**Keywords:** Research ethics, Research ethics consultation, Core competencies, Model, Clinical research, Japan

## Abstract

Research ethics consultation services (RECS), which function as an advisory service to facilitate the resolution of complex ethical issues in clinical research, have been proliferating over the last decade. However, the qualification of an individual who provides RECS, or “a research ethics consultant,” has not been thoroughly investigated, in contrast to healthcare ethics consultants, whose core competencies have been discussed and clarified to a great extent. In this study, we investigated core competencies necessary for research ethics consultants, referring to the core competency models of ethics consultants developed in the healthcare practice context, and propose a competency model for research ethics consultants.

## Introduction

Research ethics consultation services (RECS) involve consultations with researchers to provide advice and recommendations that address ethical issues (which can include issues related to society, regulations, and research fairness and integrity) related to the content of studies throughout the research period, from the clinical research protocol drafting/proposal stage before ethics review to the period after the study commences and ends. RECS first appeared in the USA and developed through most of the 1990s, and have gradually expanded in Japan as well since the late 2000s (Aizawa 2013). While RECS in the USA have consistently been led by specialists in bioethics, RECS in Japan are primarily provided by personnel responsible for the administrative office duties of ethics review committees (Iijima 2013). Currently, at least in Japan, although those personnel responsible for RECS come from a wide range of educational backgrounds and specialized fields, many of them are clerical or administrative staff who handle the general affairs of the committees, and in fact they often lack sufficient specialized education and training in research ethics (Aizawa et al. 2015; Kamisato et al. 2015). As clinical research becomes more and more advanced, specialized, and complex, however, it no longer is adequate for staff responsible for RECS to be mere clerical workers. Nor does simply being well-versed in the legal and regulatory frameworks surrounding clinical research suffice. It is therefore no longer possible to provide suitable RECS (Iijima 2013) unless a person possesses specialized knowledge and skills based on advanced education in research ethics worthy of being associated with a “profession” (Freidson 2004; Matsui 2016). Accordingly, now RECS specialists, or research ethics consultants, are required to possess a level of advanced and specialized knowledge about the vast medical field and a grasp of the principles and theories of research ethics, as well as high-level knowledge and academic experience related to debates and controversies in the field both domestically and internationally. However, there is no educational curriculum or course designed to foster research ethics consultants,^1^ and in fact, even the basic question of what constitutes the core competencies required to be a research ethics consultant has not been examined in detail in Japan or abroad.^2^ The most important challenge under these circumstances is to examine what competencies are required of the research ethics consultant to be considered a member of the profession.

The situation somewhat differs for clinical ethics consultants, whose role is to perform mediation and arbitration between parties and offer assistance or advice toward the resolution of ethics problems arising in treatment settings. Examination of the core competencies required of these consultants started earlier in the USA and the UK, where the American Society for Bioethics and Humanities (ASBH) and the UK Clinical Ethics Network (UKCEN) began developing competency models since the late 1990s (Society for Health and Human Values-Society for Bioethics Consultation 1998; American Society for Bioethics and Humanities Clinical Ethics Task Force 2009; Larcher et al. 2007; Baylis 2010). Recently, there have even been moves to implement a process of certification for clinical ethics consultants (Kodish et al. 2013). This began with the ASBH (HCEC Certification Commission n.d.), and in Japan, there is already a training and certification course administered by the Japan Association for Clinical Ethics (est. 2012).

This paper, taking as reference the foregoing discussions in healthcare ethics consultation services (HCECS), aims to consider the core competencies required of research ethics consultants and proposes a draft model. The reason for proposing such a core competencies model is related to the quality of RECS provided by research ethics consultants, whose numbers at various research institutions are likely to gradually increase in coming years. It is important to develop a shared framework that meets a set standard and to establish benchmarks for the quality of RECS and the substance of their recommendations—which will ensure that the advice and views of one institution’s RECS do not significantly differ from or clash with those of other institutions. In addition, we considered the core competencies for HCECS as a starting point because the HCECS core competencies model provides a stepping stone for considering the adjacent field of RECS, where there are currently no standards for or concrete discussions on core competencies.

### The Initiatives of AMED Research Integrity— Matsui Group (Phase I)

Briefly we describe the overview of the research group, which was a part of the research project funded by the Japan Agency for Medical Research and Development (AMED) Research and Development Program for Enhancement of Research Integrity. The primary goal of the group was to develop teaching materials for training research ethics consultants. The group is composed primarily of specialists with a wealth of experience in research and education in research ethics. The objective of the group is to develop teaching materials and educational programs for the training of “Research Ethics Education Leaders” (i.e., semi-experts/research ethics consultants) who are capable both of managing research ethics education for researchers planning and conducting clinical research at various research institutions, and of independently providing advice and consultation about research ethics. To this end, the group has been working to develop exercises for use in teaching, including a teacher’s guide (providing analysis and examples). By the end of 2018, we had developed teaching materials with 12 case studies, and held two trial training workshops for research ethics consultants using these materials in 2017 and 2018. Training workshop attendees were required to have some basic knowledge and experience in general medical ethics and bioethics and demonstrate the potential or desire to lead research ethics-related consultations and education at their institution.

This paper is based on the results of studies carried out for 27 months from January 2017 by the group. This examination of core competencies in research ethics is rooted in an awareness of the aforementioned academic and practical problems, and marks the first attempt of its kind internationally. Because the primary purpose of this project, however, was to develop teaching materials, and given the severe time constraints placed on the project, it should be stated in advance that the work remains at an exploratory stage and has not yet been thoroughly examined or verified using systematic methodology.

## Draft Model of Core Competencies

### What are “Competencies”?

While thus far we have not provided any specific definition of “competencies,” it is useful to begin by briefly reflecting on the meaning of the term. Generally, competencies refer to the basic characteristics that allow an individual to achieve effective or outstanding results in a given profession or situation based on certain standards (Spencer and Spencer 2018). Competencies are made up of both the visible elements of “skills” and “knowledge” and to some extent latent and invisible core personality elements such as self-concept and personal characteristics (Spencer and Spencer 2018; Matsushita 2011). Furthermore, because the concept of competencies was originally discovered and promoted through a comparative study of more than 200 professional fields, competencies are thought to represent characteristics shared by personnel occupying a variety of positions (Spencer and Spencer 2018). Consequently, just as in the ASBH and UKCEN reports on the core competencies of clinical ethics consultants (American Society for Bioethics and Humanities Clinical Ethics Task Force 2009; Larcher et al. 2007), it is standard to delineate the three areas of “skills,” “knowledge,” and “personal characteristics,” in order to examine the competency categories related to each area. This study followed such precedents by establishing these three domains.

### The General Flow of RECS

When considering the core competencies required of research ethics consultants who lead RECS, it is first necessary to consider some basic questions: What is the general flow by which RECS advice is formulated and implemented? What sort of responses and actions do research ethics consultants need to take and at what stage? What must be understood in advance, and what should be considered at other points during the course of RECS?

**Fig. 1:**
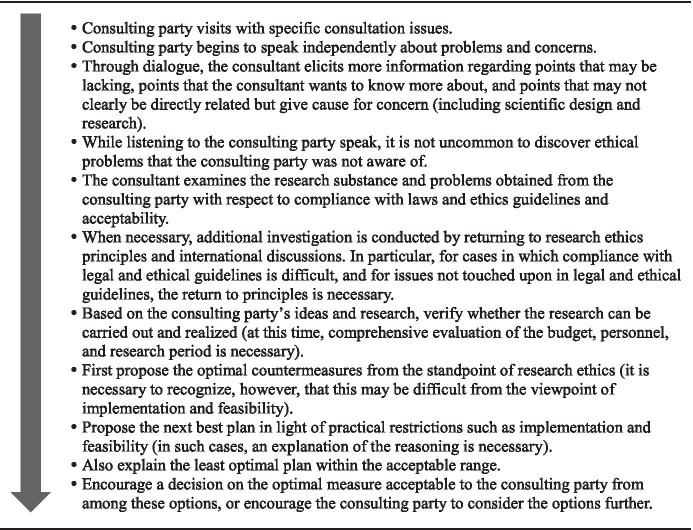
Flow of consultations common to RECS

Based on our experience (Matsui et al. 2019), the flow of RECS consultations usually proceeds in the manner shown in Fig. 1. For this reason, RECS differ from HCECS in that, as previously mentioned, RECS consultants are expected not only to be familiar with various legal and regulatory frameworks but also to already possess a fairly advanced knowledge of the vast medical field and the principles and theories of research ethics necessary to understand the contents of clinical research protocols. Without such knowledge, it would be extremely difficult to take part in research ethics consultations even if hypothetically one possessed the requisite skills.

In light of this, although many of the precedents first consider “skills,” the case of research ethics consultants responsible for RECS required that we review competencies in the order of “knowledge,” “skills,” and “personal characteristics.”

### Differences between HCECS and RECS

When evaluating the core competencies for research ethics consultants based on the model of core competencies for clinical ethics consultants, it is important to first consider the differences between HCECS and RECS.

Both HCECS and RECS share the basic function and roles of providing advice and recommendations. There are three main approaches to consultation when formulating advice and recommendations: (1) the authoritarian approach, in which the consultant unilaterally makes decisions on behalf of the concerned parties; (2) the pure facilitation approach, which simply aims to reach an agreement between parties; and (3) the ethics facilitation approach, which respects the parties’ needs and values and helps them reach decisions that do not exceed the bounds of what society regards are legally and ethically permissible (Society for Health and Human Values-Society for Bioethics Consultation 2009; Aulisio et al. 2000; Fujita and Akabayashi 2012). In HCECS, which aim to reach a consensus between two parties (doctor-patient or patient-family), the ethical facilitation approach (3) has generally been regarded as the optimal approach to achieve the best interests of patients (Fujita and Akabayashi 2012).

In RECS, however, the subject of consultation is almost always a research plan or research content, and RECS consultations very rarely directly concern human subjects. It is thus also rare for RECS to aim for mediation or consensus building between two parties. Consequently, unlike HCECS, it is not common for cases to involve urgent problems that require a swift response.^3^ Furthermore, in RECS, the main focus is placed on examining validity and performing a risk–benefit evaluation for the “group,” which in this case is the research plan or overall study content. Therefore, because the unit being examined or evaluated is not an “individual” as in the case of HCECS, examinations tend to take place from a social and macro perspective, with the dominant principle being distributive justice. In addition, problems of research ethics, compared to those in clinical ethics, often require control by external regulations such as laws and administrative guidelines (Tashiro 2011). Thus, although both RECS and HCECS are similarly expected to fulfill the function of providing advice, in the case of RECS, which lacks one of the concerned parties and must take into consideration the balance with strong external regulations, it would be difficult to adopt approach (3), let alone approach (2). Yet, because the role played by RECS differs from the review and supervisory capacity of ethics review committees, it would also be difficult to insist on the authoritarian approach (1). Thus, it is relatively common for RECS to adopt a weaker version of approach (1), which could be referred to as a “soft authoritarian approach.” Moreover, RECS, which to some degree have an authoritarian nature, also possess the hidden function of providing researchers with educational guidance about the frameworks to be followed, including external regulations, and appropriate actions to be taken in the research ethics context.

It is therefore important, when examining the core competencies of research ethics consultants, to consider the above differences between HCECS and RECS.

### Development Process and Draft Research Ethics Consultant Core Competencies Model

Following the preparatory stage outlined above, the group produced an initial draft of core competencies required of research ethics consultants. In creating the draft, the group adopted as a springboard the UKCEN 2010 model developed by Larcher et al. (2010) (Table 1), which in turn was based on the core competencies for clinical ethics consultants reported and presented by ASBH. While remaining conscious of the various similarities and differences in purpose, function/role, and authority between HCECS and RECS (Cho et al. 2018; Matsui 2016), the group first adapted the description of competencies to the context of research ethics, then repeatedly went through a process of discussing competencies within the group, making suitable revisions or modifications to necessary points, and appropriately supplementing items that appeared to be insufficient. In addition to the members of the research group, specialists in research ethics and bioethics of the Kiban-kenkyu (A) Matsui Group also participated in this process.

**Table 1:** Clinical ethics consultant core competencies, UKCEN2010 model (excerpt)

1) Knowledge	
1	Basic concepts of ethical theory and principle and the application and practice of moral reasoning
2	Knowledge of the position of the CEC in the hospital framework and links to clinical and legal governance
3	Relevant knowledge of clinical terms and disease processes
4	Cultural context of patient and staff population and of local community
5	Relevant professional codes of ethics
6	Relevant healthcare and statute law
7	Local/national government policy
2) Skills	
1	Ethical assessment skills comprise the ability to..: ・Identify and discuss the nature of the moral conflict and the need for consultation ・Elicit and understand the moral beliefs and values of all parties:
	・Analyze moral uncertainty and conflict ・Explain the ethical dimension of a case to those involved and to others ・Formulate and justify morally acceptable solutions
2	Operational and procedural skills ・Facilitation, of both case consultation discussions and CEC meetings ・Mediation and negotiation of conflict resolution in situations of emotional distress
3	Interpersonal skills ・Communication skills ・Advocacy skills to enable articulation of the views of those who find it difficult to express themselves ・Non-judgementalism, awareness of power imbalances
3) Personal characteristics ^#^	
・	Tolerance, patience and compassion
・	Honesty, fair mindedness, self-knowledge and reflection
・	Courage
・	Prudence, humility
・	Integrity

^#^The original article (Larcher et al. 2010) included descriptions of 7 matters made possible by these attributes, but this has been omitted in the present article

At the time of the 2018 workshop, participants who completed the workshop (16 people) were asked to judge the necessity/unnecessity of each competency listed in the draft model. Then, participants rated each competency based on a three-level classification—basic, advanced, or expert level requirement (Table 2). As for the 2018 workshop, the 2-day program consisted of (1) a review lecture about the basic philosophies of research ethics and the knowledge, skills, and understanding necessary for RECS (50 min); (2) a lecture about competencies (20 min); (3) simulated consultations (60 min); and (4) 4 exercises using case studies (315 min in total).

**Table 2:** Research ethics consultant level classifications

(1) Basic level (Novice Research Ethics Consultant): ・Has the minimum necessary abilities regardless of the individual's field of specialization ・Corresponds to what this research group refers to as the research ethics consultant beginner level, or to the lower level of Research Ethics Associate Expert (2) Advanced level (Intermediate Research Ethics Consultant): ・Compared to (1), has accumulated a wealth of experience (knowledge) ・To some extent can apply what they know, and handle consultations of some difficulty independently ・Capable of providing some degree of leadership and guidance to (1) ・Corresponds to what this research group refers to as the research ethics consultant intermediate level, or to the upper level of Research Ethics Associate Expert (3) Expert level (Expert Research Ethics Consultant): ・Possesses a sufficient research record in theory, principles, and policy research related to research ethics, as well as practical experience and educational experience in research ethics consulting ・Level where it is sufficiently possible to be an instructor or professor to (2), corresponding to the level of Research Ethics Expert	

Twelve participants with diverse professional backgrounds (pharmacists, general engineers, clinical research coordinators, legal staff, pharmaceutical company medical representatives, physicians, nurses, and clinical laboratory technicians) agreed to cooperate with this part of the process. Participants also self-evaluated their current level of competency immediately following the workshop (expert, 0; advanced, 1; basic, 8; below basic, 3).

Based on responses received from these 12 participants, if the views of 8 (66%) or more aligned on a given competency, it was assigned to the chosen level. For example, when 9 people agreed that the item (27)-*(5) Presentation of advice and solutions beyond the scope of regulations and guidelines* in the Domain 2–1: Ethics assessment skills was a competency necessary for being certified as an Advanced level consultant, then this item was assigned to the Advanced level requirement. Or, if the item (27)-*(5)* was considered unnecessary for being certified as a Basic level consultant by 8 people, then this item was not assigned to the Basic level requirement. When opinions regarding a certain item were divided into two (e.g., 7 vs. 5), then the level assignment was withheld. This approach was adopted to identify competencies corresponding to each level that people who are actually engaged in or who plan to engage in RECS all recognized as necessary, regardless of the aforementioned differences in professional background and self-evaluated level. For competencies that achieved an agreement of less than 8 participants, it was determined that these would be resolved through a consideration of the horizontal axis (degree) on the rubric for evaluating the education effect, which was planned for future development; for these, levels would not be classified at the current stage, and the items would be retained only as on-hold competencies (corresponding to the rubric’s vertical axis). None of the competencies included on the initial draft was judged by workshop participants to be unnecessary for research ethics consultants of any level.

Following the above-described process, the group drafted a model of core competencies required of research ethics consultants, and classified the level for each based on evaluations by the workshop participants. The results are discussed below and provided in Table 3. The UKCEN 2010 model competencies are organized around major categories of competencies in the areas (domains) of skills, knowledge, and personal characteristics, with some intermediate categories mixed in. In the draft model our group created, however, educational skill was newly added as a sub-category of skills, and everything was unified under intermediate categories. For this reason, the total number of competencies is greater than in the UKCEN model. On the other hand, while the ASBH report, for example, included a detailed examination of the content of each competency—namely, lower categories (Society for Health and Human Values-Society for Bioethics Consultation 2009)—it must be noted that our draft proposal was unable to go into that degree of detail.

**Table 3:** Proposed model of core competencies required of research ethics consultants

Competency domains and intermediate categories		Research ethics consultant level		
		Basic	Advanced	Expert
Domain 1: Knowledge				
(1)	History of research ethics, historical cases	●	●	●
(2)	Three principles of research ethics/basic theory	●	●	●
(3)	Relevant professional ethics codes (e.g., Physician Code of Ethics)	On-hold	On-hold	●
(4)	Medical research—basic design and methods	●	●	●
(5)	Domestic laws related to medical research (e.g., personal information law, clinical research law, regenerative medicine law, next-generation medical infrastructure law)	●	●	●
(6)	Primary international rules and norms for medical research (e.g., DoH, CIOMS guidelines)	-	●	●
(7)	Japanese administrative (ethical) guidelines for medical research (e.g., medical guidelines, genome guidelines)	●	●	●
(8)	Institution policies/regulations on medical research and in-facility REC/IRB, related departments (e.g., REC/IRB, clinical research support center, medical information, medical safety)	●	●	●
(9)	Basic terms and concepts related to medical research and medical care	●	●	●
(10)	Japan's medical insurance system, medical/medical policy	●	●	●
(11)	Basic matters related to research expenses (public and private)	●	●	●
(12)	Basic matters of research integrity (e.g., research misconduct, authorship)	●	●	●
(13)	Trends of domestic debates on research ethics	On-hold	On-hold	●
(14)	Trends of international debates on research ethics	-	●	●
Domain 2–1: Ethics assessment skills				
(15)	Research protocol reading skills	●	●	●
(16)	Skill of distinguishing between medical care and research	●	●	●
(17)	Skill of distinguishing legal matters from non-legal matters governed by ethical norms	●	●	●
(18)	Logical thinking/analytical skills	●	●	●
(19)	Eliciting (or understanding) the true intentions of consultees/researchers	●	●	●
(20)	Identification of ethical, legal, and social issues (ELSI) related to the consultation case: (1) Identification of problems related to the fair selection of subjects	●	●	●
(21)	Identification of ethical, legal, and social issues (ELSI) related to the consultation case: (2) Identification of problems related to risks and benefits	●	●	●
(22)	Identification of ethical, legal, and social issues (ELSI) related to the consultation case: (3) Identification of problems related to consent	●	●	●
(23)	Analysis of ELSI related to the consultation case, discussions, and presentation/explanation of advice and solutions: (1) Evaluation/judgment of risks and benefits	On-hold	On-hold	●
(24)	Analysis of ELSI related to the consultation case, discussions, and presentation/explanation of advice and solutions: (2) Presentation/explanation of grounds/reasons for justification	On-hold	On-hold	●
(25)	Analysis of ELSI related to the consultation case, discussions, and presentation/explanation of advice and solutions: (3) Discussion of other possible options/measures	-	●	●
(26)	Analysis of ELSI related to the consultation case, discussions, and presentation/explanation of advice and solutions: (4) Discovery of and pointing out hidden ELSI	-	●	●
(27)	Analysis of ELSI related to the consultation case, discussions, and presentation/explanation of advice and solutions: (5) Presentation of advice and solutions beyond the scope of regulations and guidelines	-	On-hold	●
(28)	Search and collect necessary information, supplementary information, and materials relevant to domestic situation	●	●	●
(29)	Search and collect necessary information, supplementary information, and materials relevant to international situation	-	On-hold	●
Domain 2–2: Management and procedural skills				
(30)	Dividing roles and purposes between REC/IRB review and consultation	●	●	●
(31)	Recommendations and proposals to researchers for design changes or reconsideration of research plans	On-hold	On-hold	●
(32)	Encouraging discovery of possible solutions and improvement measures on the part of researchers	-	●	●
(33)	Making rational explanations about advantages and disadvantages of each possible option	On-hold	On-hold	●
(34)	Issuing appropriate warnings to terminate, abandon, or modify issues, matters, or practices that cannot be legally or ethically permitted/justified	●	●	●
(35)	Appropriately connecting and consulting with related departments (e.g., REC/IRB, medical information, medical safety, research integrity audit office) in facility as necessary	●	●	●
(36)	Facilitating institutional organization or categorization of relevant issues or proposing improvement measures, solutions, and facility policies as necessary	-	●	●
Domain 2–3: Interpersonal skills				
(37)	General communication skills (e.g., listening, clarity, non-verbal communication)	●	●	●
(38)	Accurate and clear expression skills in Japanese language	●	●	●
(39)	Ability to first answer required questions	●	●	●
(40)	Ability to reasonably admonish researchers	-	●	●
(41)	Ability to respond with professional self-awareness and self-confidence	On-hold	On-hold	●
(42)	Ability to act with judgmentalism as necessary	On-hold	●	●
(43)	Ability to recognize power imbalance between stakeholders (e.g., consultant-researcher, researcher-subject, researcher-REC/IRB, within research team)	On-hold	On-hold	●
(44)	Ability to act neutrally and supportively without flattering authority/superiors	On-hold	On-hold	●
(45)	Ability to avoid being underestimated or abused by researchers	On-hold	On-hold	●
Domain 2–4: Educational skills				
(46)	Teaching skills	-	On-hold	●
(47)	Learning motivation/coaching skills	-	●	●
(48)	Ability to explain in plain language	●	●	●
(49)	Educational dialogue skills	On-hold	On-hold	●
(50)	Self-discipline skills	●	●	●
Domain 3: Personal characteristics				
(51)	Open-minded attitude	●	●	●
(52)	Empathic attitude	●	●	●
(53)	Neutral/independent-minded attitude, fair mindedness	●	●	●
(54)	Honesty, integrity	●	●	●
(55)	Reflective/self-knowledge attitude	●	●	●
(56)	Perseverance, diligence	●	●	●
(57)	Coherence, logicalness	●	●	●
(58)	Calmness, prudence	●	●	●
(59)	Boldness/adventurous spirit	On-hold	On-hold	●
(60)	Intellectual curiosity	On-hold	On-hold	●
(61)	Creative imagination	On-hold	On-hold	●

## Conclusion

This paper investigated the core competencies required of research ethics consultants responsible for RECS, which have proliferated in recent years, by taking as reference the existing core competencies model for clinical ethics consultants, which has already been extensively studied. The research group aimed to produce a competencies model that might lead the way globally. Although our proposed model has been developed based on solely the situation and our experiences of RECS in Japan, the core competencies which the model gives would, we consider, be also applicable to other places. It is partly because current ethical frameworks in Japan including regulations and governmental policies imitate more or less those developed in the USA which could be considered an international standard. It is also because most of ethical challenges in clinical research could be common, irrespective of one’s belonging institutions or countries, and therefore because ethical needs and requests for consultation among diverse researchers and other stakeholders including ethics review committee members would also be common, even if there were differences in regulations, policies, and research cultures. For instance, the Department of Bioethics at the U.S. National Institutes of Health (NIH) Clinical Center says that their RECS sometimes includes research participants and surrogates in consultations (Danis et al. 2012), while in Japan we unfortunately have not had such an opportunity yet to involve research participants or surrogates in our consultation services. This difference of practice in RECS might be oriented from the difference of institutional characters (i.e., all patients at the NIH Clinical Center are also research subjects, while institutionally, we have no such research-only hospital in Japan), or the difference of patient-subjects’ and/or research(-ers’) cultures between the USA and Japan. Nonetheless, we can understand actual consultation cases, their problems, and solutions experienced at the NIH, because we, too, have experienced many similar cases in Japan indeed. It accordingly implies that the core competencies required of research ethics consultants can be common globally.

The core competencies model proposed here, however, has some limitations. The model was limited to a consideration of the intermediate categories of competencies, and did not go into the detailed contents of each competency. It was also unable to go beyond an internal examination of the validity of competencies among members of our research group and collaborators. Thus, it does not reflect the consensus of the profession of individuals who specialize in teaching or studying research ethics. Furthermore, the validity of the proposed model was not verified, because the goal of this attempt was instead to provide a stepping stone for considering the necessary core competencies for research ethics consultants. The verification of validity, clarification of core competencies required of RECS supervisors, and establishment of standards and criteria represent important future tasks for the academic discipline of research ethics. Despite these limitations, the model proposed here marks the first attempt of its kind either domestically or internationally, and thus should be a significant first step toward thinking about how best to train research ethics consultants.

Another limitation of this investigation is that its scope was limited to the core competencies required of individual research ethics consultants. For this reason, the results cannot be directly applied to the competencies required of teams handling RECS, which would require a separate study. Formulating competencies for organizations will be very important for ensuring the quality of RECS in the future (Iijima 2013). Moreover, regardless of how advanced the competencies of an individual research ethics consultant may be, if for example the individual is not granted proper authority or guaranteed independence free from outside interference by the institution, they will be unable to provide advice that goes against a researcher’s will, or offer recommendations that differ from the views of the ethics review committee. Thus, rather than simply being a matter of the individual research ethics consultant’s competencies, it will also be necessary to investigate the competencies required of organizations and institutions in order to allow the individual to provide the same high-quality RECS in diverse environments, circumstances, and contexts (Matsushita 2011; OECD DeSeCo Project 2005).

## Data Availability

Available upon requests (all are in Japanese).

## Footnotes

1 The AMED Research and Development Program for Enhancement of Research Integrity Matsui Group (Phase 1) started trial workshops in late 2017 aimed at training research ethics consultants, which at least among these projects are the first in Japan.

2 While Cho et al. (2008) noted the need to consider the core competencies of research ethics consultants, in the USA, there has been little progress toward concrete studies on this topic.

3 For RECS in Japan, it seems that cases of consultations in which relatively rapid responses are required may be limited for example to emergency use of highly difficult new medical technologies and unapproved medicines for lifesaving purposes, and clinical research based on *“Advanced Medical Treatment B”* scheme or those based on *“the Evaluation System for Patient-Requested Medical Treatment”* scheme.
